# Machine learning predictive models for grading bronchopulmonary dysplasia: umbilical cord blood IL-6 as a biomarker

**DOI:** 10.3389/fped.2023.1301376

**Published:** 2023-12-15

**Authors:** Linan Gao, Pengkun Yang, Chenghan Luo, Mengyuan Lei, Zanyang Shi, Xinru Cheng, Jingdi Zhang, Wenjun Cao, Miaomiao Ren, Luwen Zhang, Bingyu Wang, Qian Zhang

**Affiliations:** ^1^ Neonatal Intensive Care Unit, The First Affiliated Hospital of Zhengzhou University, Zhengzhou, China; ^2^ Clinical Treatment and Follow-Up Center for High-Risk Newborns of Henan Province, Zhengzhou, China; ^3^ Key Laboratory for Prevention and Control of Developmental Disorders, Zhengzhou, China; ^4^ Computer Science and Technology, University of Science and Technology of China, Hefei, China; ^5^ Department of Orthopaedics, The First Affiliated Hospital of Zhengzhou University, Zhengzhou, China; ^6^ Health Care Department, The First Affiliated Hospital of Zhengzhou University, Zhengzhou, China

**Keywords:** interleukin-6, bronchopulmonary dysplasia, biomarker, machine learning, umbilical cord blood

## Abstract

**Objectives:**

This study aimed to analyze the predictive value of umbilical cord blood Interleukin-6 (UCB IL-6) for the severity-graded BPD and to establish machine learning (ML) predictive models in a Chinese population based on the 2019 NRN evidence-based guidelines.

**Methods:**

In this retrospective analysis, we included infants born with gestational age <32 weeks, who underwent UCB IL-6 testing within 24 h of admission to our NICU between 2020 and 2022. We collected their medical information encompassing the maternal, perinatal, and early neonatal phases. Furthermore, we classified the grade of BPD according to the 2019 NRN evidence-based guidelines. The correlation between UCB IL-6 and the grades of BPD was analyzed. Univariate analysis and ordinal logistic regression were employed to identify risk factors, followed by the development of ML predictive models based on XGBoost, CatBoost, LightGBM, and Random Forest. The AUROC was used to evaluate the diagnostic value of each model. Besides, we generated feature importance distribution plots based on SHAP values to emphasize the significance of UCB IL-6 in the models.

**Results:**

The study ultimately enrolled 414 preterm infants, with No BPD group (*n* = 309), Grade 1 BPD group (*n* = 73), and Grade 2–3 BPD group (*n* = 32). The levels of UCB IL-6 increased with the grades of BPD. UCB IL-6 demonstrated clinical significance in predicting various grades of BPD, particularly in distinguishing Grade 2–3 BPD patients, with an AUROC of 0.815 (95% CI: 0.753–0.877). All four ML models, XGBoost, CatBoost, LightGBM, and Random Forest, exhibited Micro-average AUROC values of 0.841, 0.870, 0.851, and 0.878, respectively. Notably, UCB IL-6 consistently appeared as the most prominent feature across the feature importance distribution plots in all four models.

**Conclusion:**

UCB IL-6 significantly contributes to predicting severity-graded BPD, especially in grade 2–3 BPD. Through the development of four ML predictive models, we highlighted UCB IL-6's importance.

## 1. Introduction

Bronchopulmonary dysplasia (BPD) is a chronic respiratory disorder originating in the neonatal period and is a major complication among premature infants. BPD results in long-term pulmonary issues, imposing significant economic burdens on both society and families (1). With advancements in neonatal care technology, more premature infants are able to survive, leading to a gradual increase in the global incidence of BPD (2). Effective treatment strategies for BPD are currently lacking (3). The prognosis for newborns with different grades of BPD varies, with a higher likelihood of mortality and neurological damage observed in those with more severe BPD (4). Assessing the severity-graded BPD usually happens late, either by 36 weeks of postnatal menstrual age (PMA) or upon discharge home. This isn't conducive for guiding treatment and devising effective strategies. Hence, early prediction of BPD grade is vital, making the exploration of predictive factors for early grading BPD crucial.

Interleukin-6 (IL-6) is a key member of the cytokine family, participating in cell-to-cell signaling and serving a crucial regulatory function in the immune system. IL-6 can accelerate the progression of lung inflammation (5). Bioinformatics analysis indicates that IL-6 is among the top ten hub genes in BPD, and both mRNA and protein levels of IL-6 are highly expressed in the peripheral blood of newborns with BPD (6). Umbilical cord blood (UCB) is non-invasive for newborns and can be collected early, making it a compelling choice for disease prediction. Previous studies have indicated a link between UCB IL-6 and the occurrence of BPD, suggesting it as a potential biomarker for predicting BPD (7–9). However, the relationship between UCB IL-6 and severity grade of BPD has yet to be investigated.

While numerous prediction tools exist for BPD, there is a scarcity of predictive models specifically addressing the severity grade of BPD (10). Most models still rely on the 2001 NICHD guidelines for assessing and grading BPD. Unfortunately, this standard is no longer applicable, failing to reflect the contemporary neonatal respiratory care practices such as the widespread use of high-flow nasal cannula (11). In 2019, a modern, evidence-based definition of BPD gained widespread recognition. This definition, established by the Neonatal Research Network (NRN), categorizes the grades of BPD based on the respiratory support patterns at PMA 36 weeks (12). When developing predictive models, artificial intelligence (AI) offers increased flexibility, allowing adaptation to diverse data types (13). AI models are particularly adept at capturing complex nonlinear relationships in data, a task that can be challenging for traditional models. In the realm of AI, machine learning (ML) is a key subfield that involves the automated identification of methods and parameters within data to find optimal solutions. Furthermore, ML models hold the potential to establish objective classification standards, thus enhancing the reliability and effectiveness of the models. ML models have emerged as promising predictive tools widely applied in clinical settings (14).

In this study, our aim is to explore the predictive value of UCB IL-6 for grading BPD and to establish effective early predictive models in a Chinese population using ML based on the 2019 NRN standards.

## 2. Materials and methods

### 2.1. Patients

**Figure 1:**
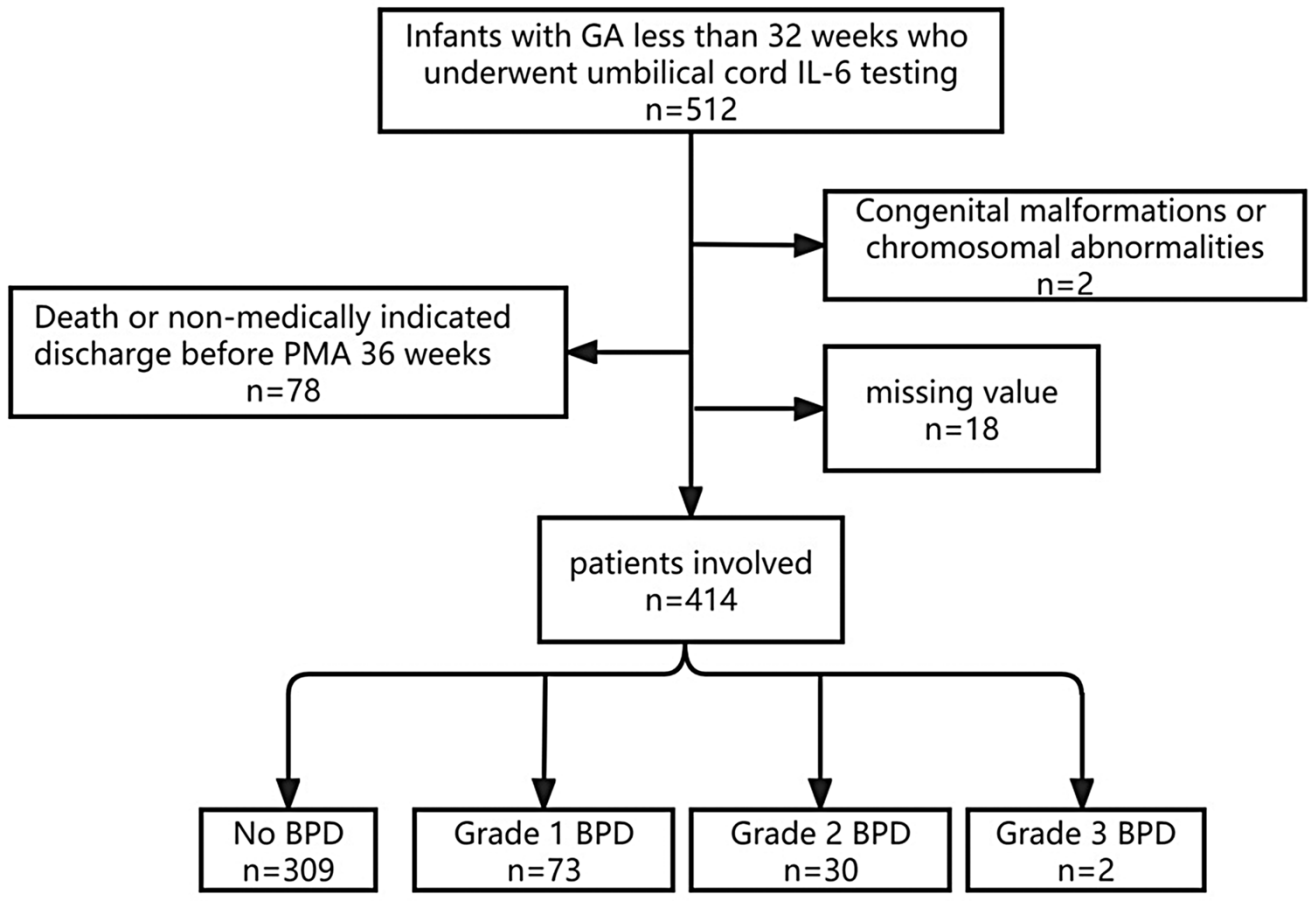
Flow chart.

We conducted a retrospective cohort study, approved by the Ethics Committee of the First Affiliated Hospital of Zhengzhou University, with the ethics approval number: 2019-KY-95. All participants provided informed consent from their parents. The study's inclusion criteria were as follows: (1) gestational age (GA) at birth of less than 32 weeks, (2) admission to the NICU between January 2020 and December 2022 with survival until PMA 36 weeks, and (3) completion of UCB IL-6 testing within 24 h of admission. Exclusion criteria encompassed (1) significant congenital anomalies or chromosomal abnormalities, (2) death or discharge against medical advice before reaching PMA 36 weeks, and (3) incomplete data. The flow chart is shown in Figure 1.

### 2.2. Data collection and UCB IL-6 determinations

We retrospectively obtained clinical data from the hospital's electronic medical record system, including maternal factors, delivery characteristics, neonatal factors, respiratory support and initial laboratory tests. Respiratory support details encompassed initial modes of continuous positive airway pressure (CPAP), initial inspiratory oxygen concentration (FiO_2_), and invasive ventilation. Initial laboratory tests covered UCB IL-6, N-terminal pro-brain natriuretic peptide (NT-proBNP), white blood cell count (WBC), pH value, chloride ion concentration, sodium ion concentration, and blood glucose levels, all collected within 24 h of admission.

The detection of UCB IL-6 concentration was performed using a multiplex microsphere flow cytometry assay (BD FACSCantoTM II flow cytometer manufactured by BD Company, Qingdao, China). The normal range for UCB IL-6 is 0–5.4 pg/ml.

Pregnancy induced hypertension is defined as systolic blood pressure of 140 mmHg or higher, diastolic blood pressure of 90 mmHg or higher, or the presence of both after 20 weeks of gestation (15). Gestational diabetes mellitus (GDM) refers to any degree of impaired glucose tolerance during pregnancy, regardless of whether diabetes was present prior to pregnancy (16). Preterm rupture of membranes (PROM) is defined as the rupture of fetal membranes before the onset of labor (17). Fetal growth restriction is defined as an estimated fetal weight or abdominal circumference below the 10th percentile for GA on ultrasound (18). According to the Fenton Chart, a birth weight (BW) at or below the 10th percentile of the average BW for the same GA is classified as small for gestational age (SGA) (19). All this data was obtained from the obstetric electronic medical records on the day of birth.

Respiratory distress syndrome (RDS) and intraventricular hemorrhage (IVH) are defined according to standard criteria (20, 21). Respiratory failure (RF) was diagnosed based on clinical features and laboratory results (22). White matter injury (WMI) diagnosis involved the use of Doppler ultrasound imaging. Hemodynamically significant patent ductus arteriosus (HsPDA) is defined as a ductal diameter of 1.5 mm or larger, and the diagnosis is confirmed through echocardiography, which shows abnormal blood flow from the aorta to the pulmonary artery (23, 24). Neonatal sepsis (NS) diagnosis includes both culture-based and culture-independent methods (25). All the aforementioned clinical complications were collected within 7 days.

### 2.3. Definition of study outcomes

#### 2.3.1. Primary outcome

In our study, the primary outcome focuses on diagnosing and grading BPD. According to the 2019 NRN evidence-based guidelines (12), severity-graded definitions of BPD are based on the mode of respiratory support at PMA 36 weeks, regardless of prior or current oxygen therapy: No BPD, no support; Grade 1 BPD, nasal cannula at flow rates ≤2 L/min; Grade 2 BPD, nasal cannula at flow rates >2 L/min or noninvasive positive airway pressure; and Grade 3 BPD, invasive mechanical ventilation. Since there were only two cases of Grade 3 BPD, we combined them with the Grade 2 BPD cases, creating a single group referred to as Grade 2–3 BPD.

#### 2.3.2. Secondary outcome

Furthermore, to explore the impact of different grades of BPD on clinical burden, we conducted supplementary analyses on length of hospital stay, total cost, and corrected age at discharge. Hospital length of stay is the entire duration from admission to discharge, measured in days. The total cost we obtained represents all expenses documented in the hospital's records during hospitalization. Corrected age at discharge was calculated by subtracting the number of weeks a child was born prematurely to her/his chronological age at the time of discharge to home (26).

### 2.4. Data analysis

For normally distributed numerical variables, we utilized mean ± SD for presentation. Conversely, for numerical variables that did not follow a normal distribution, we opted the median (P25–P75) for representation. Additionally, to meet the normal distribution assumptions, logarithmic transformations were applied to the values of UCB IL-6 and NT-proBNP using the natural logarithm (base e). Normally distributed variables were analyzed using analysis of variance (ANOVA), while non-normally distributed variables were assessed using the Kruskal-Wallis *H* test. For variables with significant *P* values, further pairwise comparisons were conducted. Categorical data were expressed as counts (%), and intergroup comparisons were performed using either the chi-square test or Fisher's exact test, as appropriate. Subsequently, the main objective was to assess the correlation between UCB IL-6 and the severity grades of BPD through logistic regression analysis.

Factors with *P* values <0.10 were selected for regression analysis. Univariate and multivariate ordinal logistic regression analyses were performed. Predictive models were established using all statistically significant risk factors (*P* < 0.05) identified through the multivariate analysis.

### 2.5. Model development and evaluation

#### 2.5.1. Model development

In the field of ML, ensemble learning algorithms are widely acknowledged as superior to traditional single-model methods. The two main branches of ensemble learning are boosting and bagging, with XGBoost, LightGBM, CatBoost, GBDT, and AdaBoost belonging to boosting algorithms, while Random Forest represents the bagging method. AdaBoost, functioning as a standalone model, is limited to binary classification and cannot be employed for multi-class tasks. Notably, XGBoost, LightGBM, and Catboost are all improved versions of GBDT. Therefore, in this study, we employed the four most commonly used methods in similar research endeavors (27–29): XGBoost, LightGBM, Catboost, and RF.

Ten-fold cross-validation was utilized to train and evaluate our four models. The dataset was split into ten uniform partitions using stratified sampling, maintaining consistent proportions for each category within every partition, mirroring the original dataset. Each model underwent ten rounds of training and evaluation, with nine subsets used for training and one subset for testing in each iteration. This tenfold process ensured each subset was used for testing once. The final evaluation represented the average of these ten assessment rounds, providing a more stable assessment of model performance by reducing the impact of random data partitioning. To address the imbalance in sample sizes among different classes in our dataset, we applied balanced sampling to maintain consistent training across all categories. Additionally, we conducted a grid hyperparameter search to optimize the models' performance.

#### 2.5.2. Model evaluation

We used Receiver Operating Characteristic (ROC) curves and Area Under the Curve (AUC) to assess the models’ classification performance. We not only utilized Macro-average ROC for an overall evaluation but also employed Micro-average ROC to mitigate class imbalance, offering a more accurate reflection of the models' predictive capabilities. Apart from these metrics, we also utilized F1 score, precision, recall and accuracy to assess the models' performance. Furthermore, we calculated SHAP values and generated feature importance distribution plots to evaluate the significance of UCB IL-6 in each model.

#### 2.5.3. Experiment setting

All our experiments were based on Python 3.8, utilizing the following libraries: scikit-learn 0.24.1, XGBoost, LightGBM, Catboost, and GridSearch. We set the random seed to 42. The trained models and inference evaluation codes have been uploaded to GitHub. You can click the following link to view them (https://github.com/1183371714/GBD).

## 3. Results

### 3.1. Demographics and clinical burden

The research ultimately included 414 preterm infants, with merely two cases falling into the Grade 3 BPD category. All patients were divided into three groups: the No BPD group (*n* = 309), the Grade 1 BPD group (*n* = 73), and the Grade 2–3 BPD group (*n* = 32). The demographic characteristics and clinical burden of the patients are detailed in Table 1. It's clear that infants with more severe BPD tend to have a lower GA, and those in the No BPD group have the highest BW. Additionally, Grade 2–3 BPD infants have a higher proportion of males compared to the No BPD group. Notably, the analysis of differences in clinical burdens among the three groups indicates that groups with higher BPD grades experience heavier clinical burdens.

**Table 1:** Demographic and clinical burden in different groups.

Variables	No BPD (*n* = 309)	Grade 1 (*n* = 73)	Grade 2–3 (*n* = 32)	*P*
GA, week, median (P25, P75)	30.3 (29.2, 31.2)*,**	30.0 (28.6, 30.6)***	28.5 (27.4, 29.5)	<0.001****
BW, kg, mean ± SD	1.38 ± 0.31*,**	1.21 ± 0.26	1.11 ± 0.25	<0.001****
Male, *n* (%)	151 (48.9)**	40 (54.8)	24 (75.0)	0.016****
SGA, *n* (%)	34 (60.7)*	17 (30.4)	5 (8.9)	0.023****
Length of hospital stay, day, median (P25, P75)	41.0 (32.0, 54.0)*,**	55.0 (45.0, 72.0)***	85.5 (62.5, 101.0)	<0.001****
Corrected age at discharge, week, median (P25, P75)	36.2 (35.4, 37.2)*,**	37.6 (36.5, 38.5)***	40.2 (38.15, 42.1)	<0.001****
Total cost, thousand yuan, mean ± SD	100.65 ± 42.5*,**	146.08 ± 54.60***	239.60 ± 132.69	<0.001****

GA, gestational age; BW, birth weight; SGA, small-for-gestational-age; SD, standard deviation; P10, 10th percentile.

* *P *< 0.05, comparison between No BPD and Grade 1.

** *P *< 0.05, comparison between No BPD and Grade 2–3.

*** *P *< 0.05, comparison between Grade 1 and Grade 2–3.

**** Comparison between the groups with significance.

In the univariate analysis, infants with Grade 2–3 BPD were less likely to receive antenatal corticosteroid treatment. After birth, significant differences were observed among the different groups in Apgar scores and rates of asphyxia. Infants with a higher grade of BPD exhibited lower Apgar scores and a greater likelihood of requiring resuscitation for asphyxia. The risk factors during the antepartum and perinatal periods are listed in Table 2.

**Table 2:** Antenatal and birth characteristics in the patients among three groups.

Characteristics	No BPD (*n* = 309)	Grade 1 (*n* = 73)	Grade 2–3 (*n* = 32)	*P*
Maternal age, year, mean ± SD	31.27 ± 4.85	32.27 ± 5.47	31.41 ± 4.99	0.299
Multiple pregnancy, *n* (%)	72 (23.3)	13 (17.8)	9 (28.1)	0.450
Embryo transfer, *n* (%)	50 (16.2)	16 (21.9)	6 (18.8)	0.497
History of abnormal pregnancy, *n* (%)	168 (54.4)	39 (53.4)	17 (53.1)	0.983
Antenatal tocolytics, *n* (%)	156 (50.5)	34 (46.6)	11 (34.4)	0.207
Prenatal steroids, *n* (%)	166 (53.7)**	32 (43.8)	7 (21.9)	0.002****
Gestational hypertension, *n* (%)	116 (37.5)	26 (35.6)	14 (43.8)	0.727
GDM, *n* (%)	73 (23.6)	14 (19.2)	7 (21.9)	0.712
IUGR, *n* (%)	44 (14.2)	8 (11)	2 (6.3)	0.448
Fetal distress, *n* (%)	76 (24.6)	17 (23.3)	7 (21.9)	0.940
PROM, *n* (%)	98 (31.7)	17 (23.3)	9 (28.1)	0.358
Cesarean delivery, *n* (%)	244 (79)	61 (83.6)	25 (78.1)	0.662
Placental abnormalities, *n* (%)	75 (24.3)	22 (30.1)	9 (28.1)	0.554
Umbilical cord abnormalities, *n* (%)	85 (27.5)	25 (34.2)	10 (31.3)	0.499
Meconium-stained amniotic fluid, *n* (%)	55 (17.8)	10 (13.7)	9 (28.1)	0.206
1-min Apgar score, median (P25–P75)	8.0 (7.0, 9.0)**	8.0 (7.0, 9.0)	7.5 (5.0, 8.0)	<0.001****
5-min Apgar score, median (P25–P75)	9.0 (9.0, 10.0)**	9.0 (8.0, 10.0)***	8.0 (7.0, 9.0)	<0.001****
Perinatal asphyxia, *n* (%)	72 (23.3)**	26 (35.6)	14 (43.8)	0.009****

GDM, gestational diabetes mellitus; IUGR, intrauterine growth restriction; PROM, premature rupture of membranes; SD, standard deviation; P25–P75, percentiles 25–75.

** *P *< 0.05, comparison between No BPD and Grade 2–3.

*** *P *< 0.05, comparison between Grade 1 and Grade 2–3.

**** Comparison between the groups with significance.

Patients with No BPD initially require the lowest FiO_2_ and are most likely to receive initial breathing support through CPAP. Grade 2–3 BPD patients require the highest FiO_2_ and are most likely to receive initial support through invasive ventilation. Compared to the No BPD group, Grade 2–3 BPD patients are more likely to receive surfactant therapy. Among the three groups, Grade 2–3 BPD patients have the highest levels of initial NT-proBNP and UCB IL-6, while having the lowest pH values. Children in the No BPD group have the lowest likelihood of experiencing pulmonary hemorrhage and HsPDA. The more severe the condition, the higher the likelihood of RF, pulmonary hypertension, and IVH. The early postnatal clinical characteristics are presented in Table 3.

**Table 3:** Early postnatal characteristics among three groups.

Variables	No BPD (*n* = 309)	Grade 1 (*n* = 73)	Grade 2–3 (*n* = 32)	*P*
Laboratory test (initial)				
LnIL-6, pg/ml, mean ± SD	2.80 ± 1.90*,**	3.30 ± 1.92***	4.88 ± 1.28	<0.001****
LnNT-proBNP, ng/ml, mean ± SD	8.00 ± 1.14**	8.32 ± 1.12	8.54 ± 1.07	<0.001****
WBC, ×10^9^/L, median (P25–P75)	7.09 (5.38, 9.76)	7.31 (4.74, 9.54)	7.34 (5.49, 11.8)	0.67
PH, mean ± SD	7.27 ± 0.09**	7.26 ± 0.11	7.2 ± 0.10	<0.001****
Glucose, mmol/L, median (P25–P75)	3.4 (2.62, 4.5)	3.7 (2.98, 4.79)	3.83 (2.56, 4.62)	0.275
Cl, mmol/L, median (P25–P75)	106 (104, 108)	106 (103.5, 107.9)	108 (103.6, 110.7)	0.078
Na, mmol/L, mean ± SD	135.30 ± 2.70	135.43 ± 2.93	135.23 ± 3.43	0.927
Complications				
RDS, *n* (%)	298 (96.4)	72 (98.6)	31 (96.9)	0.699
HsPDA, *n* (%)	27 (8.7)*,**	24 (32.9)	11 (34.4)	<0.001****
RF, *n* (%)	77 (24.9)*,**	33 (45.2)	20 (62.5)	<0.001****
Pulmonary hypertension, *n* (%)	10 (3.2)**	4 (5.5)	4 (12.5)	0.043****
Pulmonary hemorrhage, *n* (%)	7 (2.3)*	7 (9.6)	3 (9.4)	0.004****
IVH, *n* (%)	167 (54)*,**	53 (72.6)	25 (78.1)	0.001****
WMI, *n* (%)	257 (83.8)	67 (91.8)	27 (84.4)	0.178
NS, *n* (%)	95 (30.7)	31 (42.5)	12 (37.5)	0.134
Respiratory support and treatment				
Initial FiO_2_, %, median (P25–P75)	30 (25, 32)*,**	30 (30, 40)	40 (30, 43)	<0.001****
Initial CPAP, *n* (%)	174 (56.3)*,**	21 (28.8)	5 (15.6)	<0.001****
Invasive ventilation, *n* (%)	44 (14.2)*,**	24 (32.9)	18 (56.3)	<0.001****
Caffeine, *n* (%)	217 (70.2)	59 (80.8)	28 (87.5)	0.031****
Surfactant therapy, *n* (%)	167 (54)**	50 (68.5)	26 (81.3)	<0.001****

LnIL-6, ln interleukin-6; NT-proBNP, N-terminal pro-brain natriuretic peptide; WBC, white blood cell counts; PH, pH value; Cl, chloride concentration; Na, sodium concentration; RDS, respiratory distress syndrome; HsPDA, hemodynamically significant patent ductus arteriosus; RF, respiratory failure; IVH, intraventricular hemorrhage; WMI, white matter injury; NS, neonatal sepsis; FiO_2_, fraction of inspiration O_2_; CPAP, continuous positive airway pressure; SD, standard deviation; P25–P75, percentiles 25–75.

* *P *< 0.05, comparison between No BPD and Grade 1.

** *P *< 0.05, comparison between No BPD and Grade 2–3.

*** *P *< 0.05, comparison between Grade 1 and Grade 2–3.

**** Comparison between the groups with significance.

### 3.2. The predictive value of UCB IL-6 levels for the severity grade of BPD

**Figure 2:**
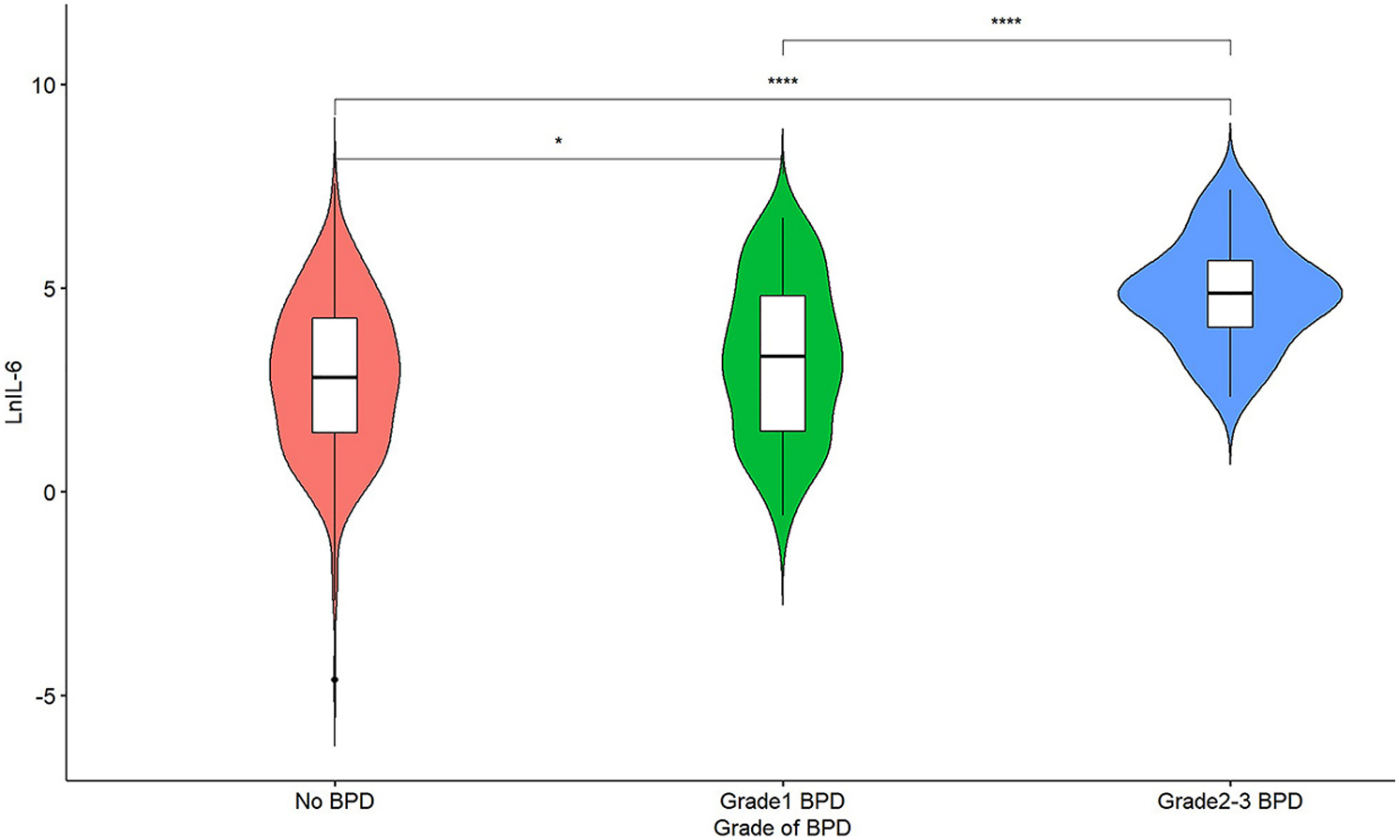
A violin plot of lnIL-6 levels among different grade groups of BPD; *represents *P* < 0.05, and ****represents *P* < 0.0001.

The comparison of UCB IL-6 levels among different BPD groups is illustrated in Figure 2. Significant differences (*P* < 0.05) were observed between any two groups, with higher UCB IL-6 levels in the groups with more severe BPD.

**Figure 3:**
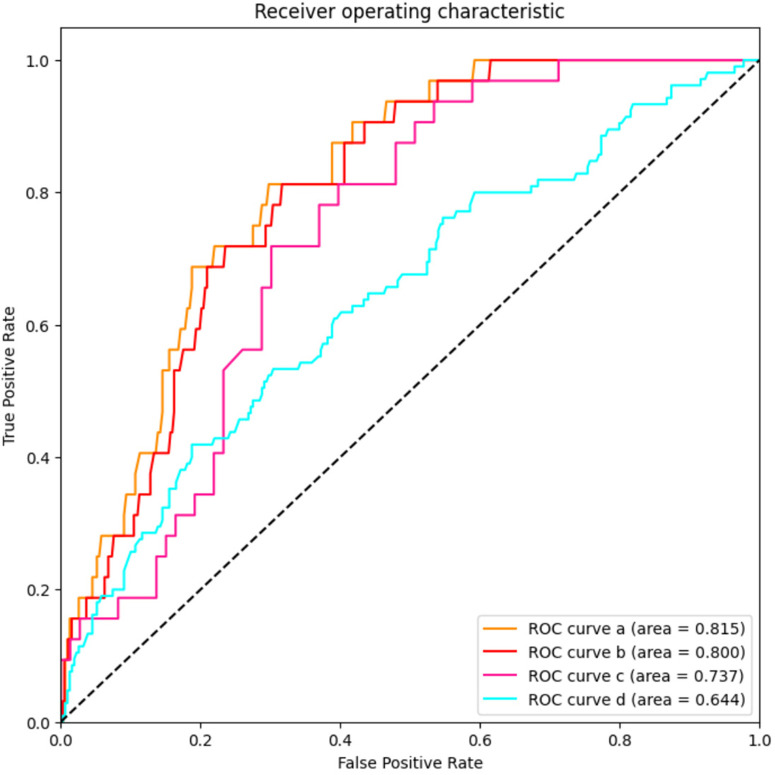
The predictive value of umbilical cord blood IL-6 levels for the grade of BPD. AUC stands for area under the receiver operating characteristic curve; and BPD stands for bronchopulmonary dysplasia. ROC curve a: LnIL-6 levels for distinguishes infants with Grade 2–3 BPD from those with No BPD; ROC curve b: LnIL-6 levels for distinguishing between less severe (Grade 0–1) and more severe (Grade 2–3) BPD; ROC curve c: LnIL-6 levels’ predictive value for higher-grade BPD (Grade 2–3 BPD) in patients with BPD; ROC curve d: LnIL-6 levels for prediction of BPD occurrence.

UCB IL-6 levels were not effective in distinguishing between infants with Grade 1 BPD and those with No BPD. However, as illustrated in Figure 3, in ROC curve a, UCB IL-6 effectively distinguishes infants with Grade 2–3 BPD from those with No BPD, with an AUC of 0.815 (95% CI: 0.753–0.877). In ROC curve b, UCB IL-6 demonstrates predictive potential for distinguishing between less severe (Grade 0–1) and more severe (Grade 2–3) BPD, supported by an AUC of 0.800 (95% CI: 0.738–0.862). The optimal cutoff value for UCB IL-6 in both cases was 47.70 pg/ml. The sensitivity was 81.3% in both cases, with specificities of 70.2% and 68.3%, respectively. Within the subgroup of BPD patients, ROC curve c demonstrates UCB IL-6's predictive value for higher-grade BPD (Grade 2–3 BPD) with an AUC of 0.737 (95% CI: 0.642–0.831). The optimal cutoff value was 76.71 pg/ml, providing a sensitivity of 71.9% and specificity of 69.9%. In ROC curve d, the ROC curve demonstrates the predictive ability of UCB IL-6 for BPD occurrence. The AUC was calculated as 0.644 (95% CI: 0.582–0.706), with an optimal cutoff value of 90.92 pg/ml. Sensitivity and specificity were 41.9% and 81.2%, respectively.

### 3.3. Multivariable analysis

The results of the ordinal logistic regression analysis are presented in Table 4. Patients were categorized based on severity grade as follows: No BPD as 0, Grade 1 BPD as 1, and Grade 2–3 BPD as 2. According to the logistic regression analysis, UCB IL-6, NT-proBNP, pH value, Apgar scores at 1 and 5 min, perinatal asphyxia, initial FiO_2_, invasive ventilation, surfactant therapy, HsPDA, RF, and IVH were identified as independent risk factors for grading BPD.

**Table 4:** Results of the ordinal logistic regression model for severity-graded BPD.

Characteristics	Crude odds ratio^a^ 95% CI	*P*^a^ value	Adjusted odds ratio^b^ 95% CI	*P*^b^ value
IL-6	1.369 (1.208, 1.551)	<0.001	1.343 (1.179, 1.530)	<0.001
NT-proBNP	1.370 (1.120, 1.675)	0.002	1.251 (1.010, 1.548)	0.040
PH	0.042 (0.004, 0.420)	0.007	0.075 (0.007, 0.807)	0.033
1-min Apgar score	0.794 (0.715, 0.882)	<0.001	0.833 (0.745, 0.932)	0.001
5-min Apgar score	0.634 (0.541, 0.745)	<0.001	0.689 (0.583, 0.814)	<0.001
Perinatal asphyxia	2.052 (1.287, 3.274)	0.003	1.806 (1.102, 2.956)	0.019
FiO_2_	1.051 (1.033, 1.070)	<0.001	1.030 (1.012, 1.050)	0.001
Invasive ventilation	4.306 (2.638, 7.029)	<0.001	2.933 (1.694, 5.083)	<0.001
Surfactant therapy	2.286 (1.413, 3.699)	0.001	1.756 (1.058, 2.915)	0.029
HsPDA	4.735 (2.762, 8.125)	<0.001	4.289 (2.450, 7.508)	<0.001
RF	3.180 (2.018, 5.008)	<0.001	2.462 (1.523, 3.979)	<0.001
IVH	2.467 (1.513, 4.023)	<0.001	2.121 (1.273, 3.540)	0.004

IL-6, ln (interleukin-6); NT-proBNP, N-terminal pro-brain natriuretic peptide; PH, pH value; FiO_2_, fraction of inspiration O_2_; HsPDA, hemodynamically significant patent ductus arteriosus; RF, respiratory failure; IVH, intraventricular hemorrhage; 95% CI, 95% confidence interval.

^a^ Crude odd ratio and *P* values are of ordinal logistic regression.

^b^ Adjusted odds ratios and *P* value for gestational age, gender and birth weight.

### 3.4. Model performance and feature importance distribution

**Figure 4:**
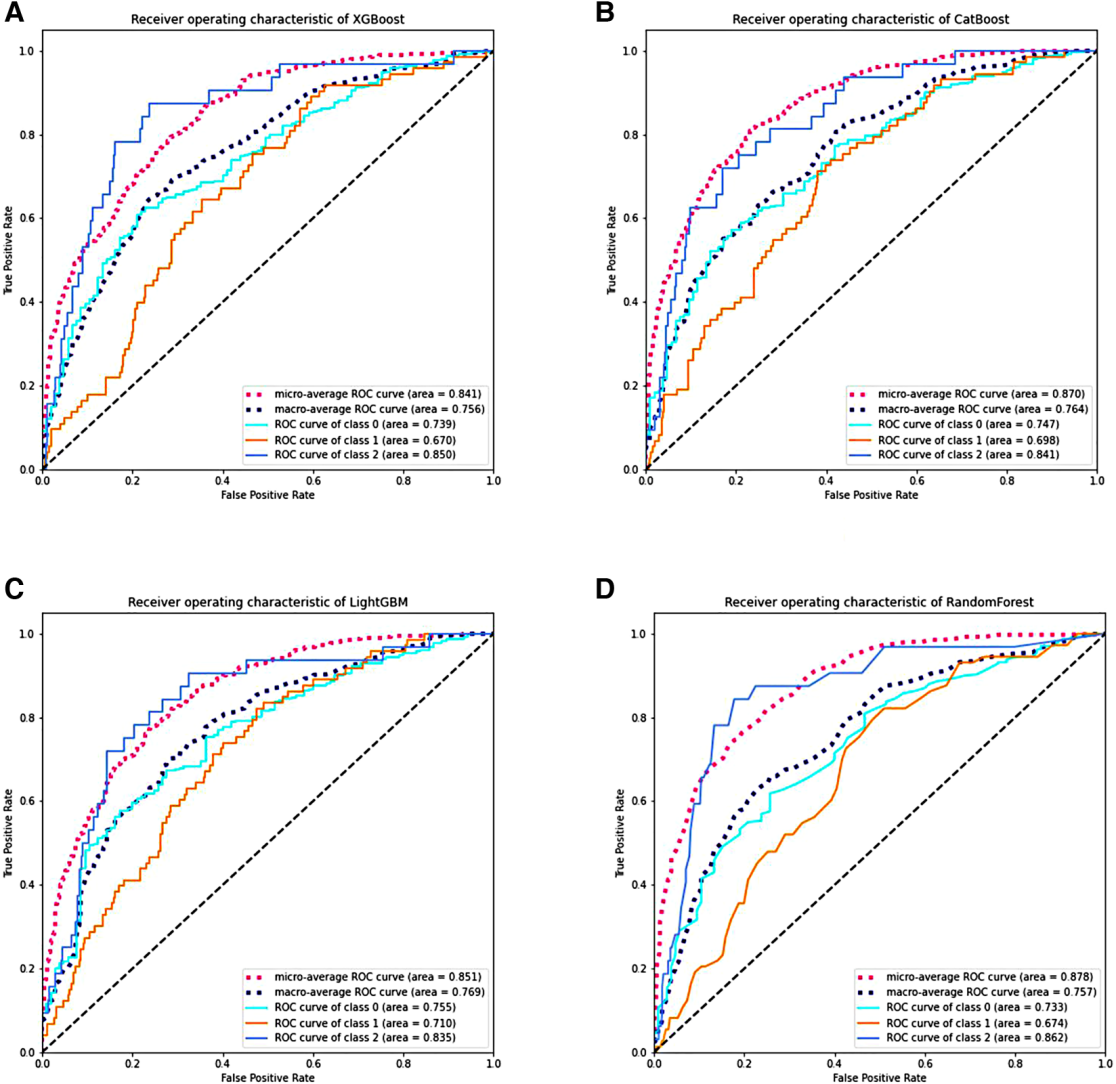
The performance of various machine learning models through ten-fold cross-validation. (**A–D**) Display the ROC curves of the XGBoost, CatBoost, LightGBM, and RF models. Class 0 represents No BPD; class 1 represents Grade 1 BPD, and class 2 represents Grade 2–3 BPD. RF stands for random forest, ROC stands for receiver operating characteristic, AUC stands for area under the receiver operating characteristic curve, and BPD stands for bronchopulmonary dysplasia.

Twelve clinical factors with significant differences in multivariate analysis, as well as key factors (gender, GA and BW), were incorporated into ML models. ROC curves were derived through ten-fold cross-validation. The Micro-average AUROC values for the XGBoost, CatBoost, LightGBM, and Random Forest models were 0.841, 0.870, 0.851, and 0.878, respectively (Figure 4). Detailed metrics for these four models can be found in Supplementary Table 1.

**Figure 5:**
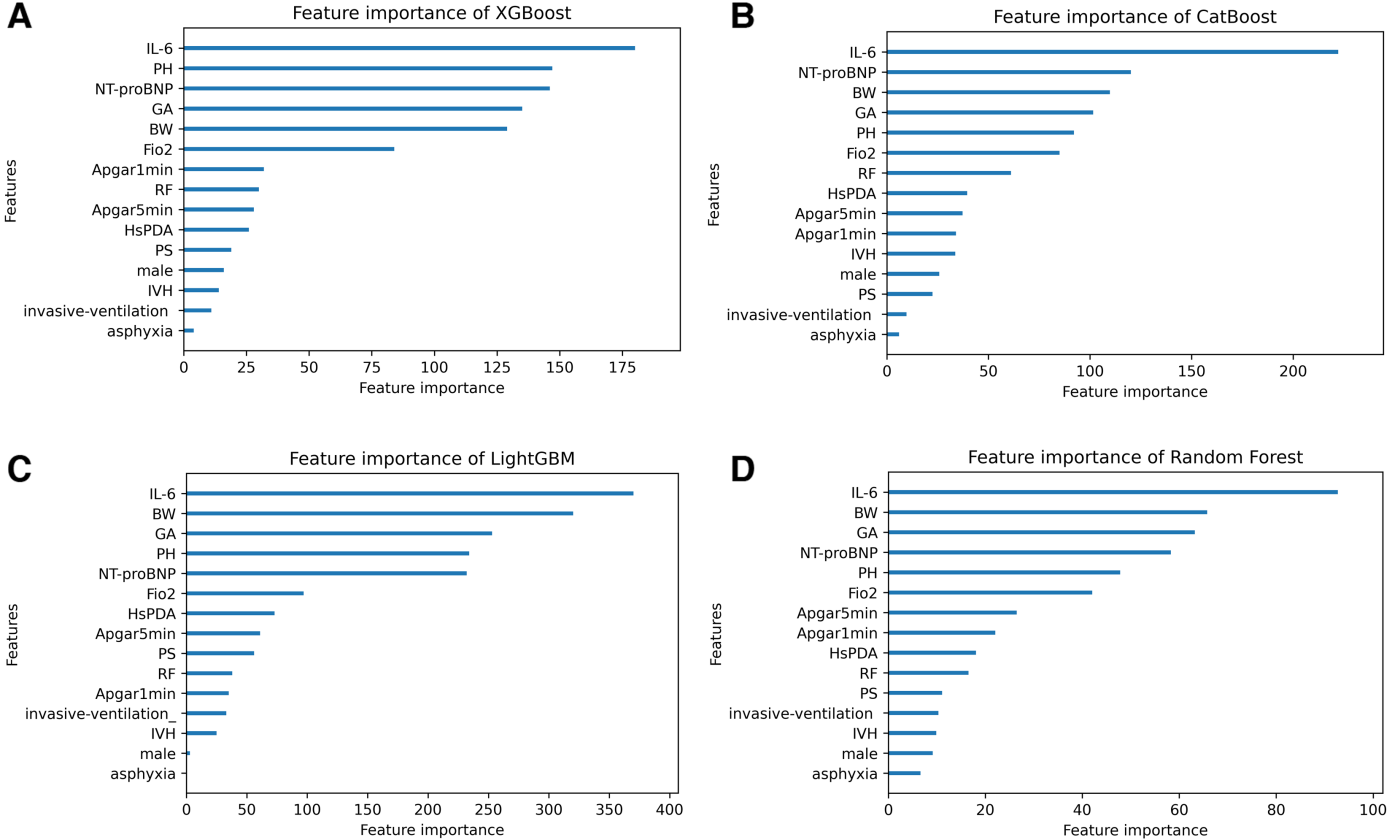
The feature importance distribution charts of four machine learning models. (**A–D**) Display the feature importance distribution charts of the XGBoost, CatBoost, LightGBM, and RF models. IL-6 umbilical cord blood interleukin-6, BW birth weight, GA gestational age, PH pH value, FiO_2_ fraction of inspiration O_2_, RF respiratory failure, HsPDA hemodynamically significant patent ductus arteriosus, IVH intraventricular hemorrhage, PS pulmonary surfactant.

Based on SHAP values, Figure 5 illustrates the feature importance distribution for each model. It is evident that UCB IL-6 is the most crucial feature across all models.

## 4. Discussion

We verified the predictive significance of UCB IL-6 for severity-graded BPD among a Chinese cohort, in accordance with the 2019 NRN guidelines. Based on the results of univariate analysis, UCB IL-6 levels exhibited an increase in tandem with the grades of BPD. Our study demonstrated that UCB IL-6 predicts the occurrence of BPD. Additionally, it predicts the presence of Grades 2–3 BPD when compared to either No BPD or Grade 1 BPD. What's more, ordinal logistic regression analyses indicated that UCB IL-6 is a predictive factor for grading BPD. UCB IL-6 predicts Grade 2–3 BPD better than Grade 1 BPD, as do the four ML models we developed. It was observed that the UCB IL-6 factor consistently ranked as the top feature in the feature importance distribution charts of the four models.

The 2001 NICHD criteria for BPD are outdated due to advancements in respiratory support methods. The 2018 NICHD criteria appear to be somewhat complex. The 2019 NRN guidelines are easy and effective, and using them to assess the severity-graded BPD can provide the most accurate predictions regarding infant outcomes at 18–26 months of corrected age (12). Based on the 2019 NRN standards, it was found that infants classified with a higher grade BPD have a significantly higher risk of requiring tracheostomy or facing mortality compared to those with a lower grade of BPD (30). Early prediction of severity-graded BPD under the 2019 NRN standards is necessary, as it can predict clinical burden, guide treatment, and inform follow-up strategies.

Yan et al. (6) found that the higher the grade of BPD, the higher the concentration of peripheral blood IL-6. Cansu Yılmaz et al. (31) found that elevated levels of IL-6 in tracheal aspirates of newborns were associated with the severity grade of BPD. In contrast, our study focuses on UCB, obtained at an earlier time point, and poses lower invasiveness to the neonates. IL-6 may play a pivotal role in the pathogenesis of BPD. One possible explanation is that activation of IL-6 signaling in macrophages by high oxygen impairs the homeostasis of alveolar type II epithelial cells and disrupts the formation of elastic fibers, thereby inhibiting lung growth (32). Additionally, studies suggest that the crosstalk between inflammation and cell death might be associated with oxygen-induced lung injury in BPD. Future therapeutic approaches for BPD should be centered on suppressing the expression of cytokines (33). Zhang et al. (34) revealed that BPD might be influenced by the inflammatory response to the gut microbiota. Further in-depth research and exploration are necessary to elucidate the specific underlying mechanisms.

Most predictive models traditionally relied on statistical methods. However, there is a recent surge in utilizing AI techniques to develop BPD prediction models. For example, a study integrated clinical data and genomics to construct a ML model for predicting BPD and severe BPD, achieving an AUC of 0.872 (35). In 2023, Wen He and colleagues developed multiple ML models for predicting BPD severity using clinical data, with the highest AUC reaching 0.86 (10). However, it's important to note that their criteria for diagnosing and grading BPD were based on the 2001 NICHD guidelines, which might not fully account for advances in respiratory support technology today, limiting the practical use of their models. In contrast, our models adhere to the more up-to-date 2019 NRN standards, making them more suitable for clinical application. Furthermore, our models include new predictive factors—NT-proBNP and UCB IL-6. Notably, UCB IL-6, being non-invasive, consistently held the top rank in the feature importance distribution charts within all four models, signaling its significance.

Other than UCB IL-6, we observed that in our four models, the top five factors in terms of importance also include BW, GA, NT-proBNP, pH value, and FiO_2_. Factors that impede alveolar maturation, such as BW and GA, are considered crucial elements in the pathology of BPD. Previous studies have demonstrated that NT-proBNP levels can predict moderate-to-severe BPD or mortality (36–38). Given that, in this study, based on the 2019 NRN criteria, NT-proBNP remains a significant risk factor for grading BPD, further attention should be directed towards understanding how NT-proBNP influences infant lung development. According to our study, timely correction of premature infants' pH levels and prevention of acidosis are crucial. Additionally, we noticed high initial FiO_2_ levels as an independent risk. One possible reason is that elevated oxygen triggers reactive oxygen species, harming the lungs (39).

However, our study has limitations. The small number of patients with Grade 3 BPD in our level III NICU forces us to combine it with Grade 2 BPD, representing a more severe condition. Therefore, we are unable to predict the likelihood of an infant developing grade 3 BPD specifically; instead, we can only predict whether the newborn is at a more severe level of BPD. Being retrospective, confounding factors weren't controlled. Additionally, our data were collected from a single center, potentially not representing all patients. Future research requires a prospective study with a larger sample size, multiple centers, and external validation to incorporate UCB IL-6 in predicting preterm infant outcomes.

## 5. Conclusion

Our research focused on preterm infants with GA less than 32 weeks. We developed four ML models and found that UCB IL-6 plays a crucial role in predicting the severity grade of BPD.

## Data Availability

The raw data supporting the conclusions of this article will be made available by the authors, without undue reservation.
