# Supplementary material for: Machine learning predictive models for grading bronchopulmonary dysplasia: umbilical cord blood IL-6 as a biomarker
Source: Front Pediatr. 2023 Dec 15;11:1301376. doi: 10.3389/fped.2023.1301376 (PMC10757373; doi:10.3389/fped.2023.1301376)
Supplement: Supplementary file 1 [file Table1.docx]

Supplementary Material

Supplementary Table 1. The detail indexes for all the machine learning models

|  |  | Precision | Recall | F1 score | Accuracy |
| --- | --- | --- | --- | --- | --- |
| XGBoost | No BPD | 0.725 | 0.836 | 0.776 | 0.725 |
|  | Grade 1 BPD | 0.315 | 0.250 | 0.279 | 0.315 |
|  | Grade 2-3 BPD | 0.500 | 0.296 | 0.372 | 0.500 |
|  | Total | 0.605 | 0.635 | 0.613 | 0.635 |
| CatBoost | No BPD | 0.819 | 0.819 | 0.819 | 0.819 |
|  | Grade 1 BPD | 0.301 | 0.344 | 0.321 | 0.301 |
|  | Grade 2-3 BPD | 0.438 | 0.341 | 0.384 | 0.438 |
|  | Total | 0.701 | 0.698 | 0.699 | 0.698 |
| LightGBM | No BPD | 0.761 | 0.855 | 0.805 | 0.761 |
|  | Grade 1 BPD | 0.411 | 0.361 | 0.385 | 0.411 |
|  | Grade 2-3 BPD | 0.500 | 0.286 | 0.364 | 0.500 |
|  | Total | 0.655 | 0.679 | 0.661 | 0.679 |
| Random Forest | No BPD | 0.854 | 0.802 | 0.828 | 0.854 |
|  | Grade 1 BPD | 0.219 | 0.271 | 0.242 | 0.219 |
|  | Grade 2-3 BPD | 0.281 | 0.346 | 0.310 | 0.281 |
|  | Total | 0.728 | 0.698 | 0.712 | 0.698 |

BPD：bronchopulmonary dysplasia
